# Gender disparities in coronary artery disease: a review of factors influencing clinical outcomes

**DOI:** 10.1007/s12471-025-01996-7

**Published:** 2025-11-10

**Authors:** Arsalan Siddiqui, Randeep Gill, Marc Ringor, Jasmine K. Dugal, Arpinder Malhi, Ala Abdallah, Dalia Hawwass, Nazanin Houshmand, Tahir Tak

**Affiliations:** 1 Department of Internal Medicine, Kirk Kerkorian School of Medicine at UNLV, Las Vegas, NV USA; 2 Department of Cardiovascular Medicine, Kirk Kerkorian School of Medicine at UNLV, Las Vegas, NV USA

**Keywords:** Coronary heart disease, Health disparities, Gender disparities, Risk factors, Myocardial ischemia

## Abstract

**Background:**

Coronary artery disease (CAD) is a leading cause of morbidity and mortality worldwide, affecting both women and men. However, its evaluation and treatment have historically been influenced by gender, resulting in significant disparities for women.

**Objective:**

This review aims to comprehensively examine the literature on gender disparities in the care of patients with CAD.

**Results:**

Evidence highlights several key inequalities, including the relatively greater impact of shared risk factors in women, the presence of female-specific risk factors, differences in CAD symptom presentation, and reduced screening sensitivity and management quality in women compared with men.

**Conclusions:**

Addressing these disparities requires updated screening strategies that recognize the unique clinical manifestations of CAD in women, increasing awareness among both women and healthcare providers, greater inclusion of women in CAD research studies, revisiting the role of hormonal replacement therapy, and integrating emerging tools such as genetic research and artificial intelligence. These steps have the potential to improve the equity and effectiveness of CAD management across genders.

## Introduction

**Fig. 1:**
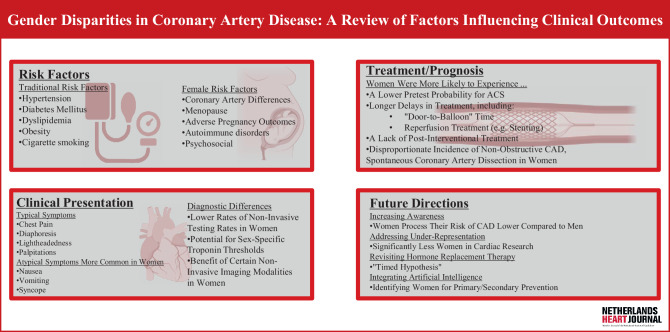
Infographic of Study

Coronary artery disease (CAD) is one of the most prevalent medical conditions worldwide, and despite advancements in its recognition and treatment, women remain at a significantly greater risk of suffering complications and death than men [1]. There are multiple factors contributing to this variation, including biological differences, comorbid status, disease perception, delays in receiving appropriate care, and historic underrepresentation. Moreover, factors that increase susceptibility to CAD, including uncontrolled hypertension and diabetes mellitus (DM), continue to be underdiagnosed and under-treated in females [2]. Recent studies have also found that while the incidence of myocardial infarction (MI) remains similar in men, the past two decades have seen women increasingly develop MI at younger ages [2]. Given this disparity, cardiologists and advocacy groups have collaborated to understand the factors leading to suboptimal outcomes in female patients. This review aims to comprehensively investigate the literature on gender disparities in the care of CAD patients. While we acknowledge that gender exists on a spectrum, in this review, we use the term “gender” as an umbrella term referring to categorical distinctions between the male (men) and female (women) sexes (Fig. 1).

### Shared risk factors

While CAD shares many risk factors across genders, it is now well recognized that they influence outcomes differently. Hypertension is more prevalent in women with MI, and a higher systolic blood pressure in women confers a greater relative risk of MI than in men [3]. Similarly, DM raises the risk of incident coronary heart disease by 44% compared to men with DM [4]. Dyslipidemia is also a significant risk factor, and hormonal changes, particularly after the menopausal transition, accelerate atherosclerosis progression [5]. Obesity, a well-established modifiable risk factor for hypertension and DM, has also been shown to confer an excess cardiovascular disease (including CAD) risk in women [6]. Although global cigarette consumption has decreased worldwide, a recent meta-analysis found that even low consumption of cigarettes carries a higher relative risk of STEMI in women compared to men [7]. Similarly, a systematic review involving 22 million individuals found that lower socioeconomic markers, such as education level and income, are associated with a greater coronary heart disease risk in women. In particular, lower education levels confer a significant relative risk of 1.34 for coronary heart disease in women compared to men [8]. While several European studies examining the intersection of gender and race in CAD disparities are lacking, data from the United States reveal that Black women have higher non-fatal CAD rates than White women [9]. Given these risk factors’ greater impact on women, strategies should address both prevalence reduction and systemic biases related to race and socioeconomic status. Additionally, future research should examine whether stricter disease targets may be warranted for women.

### Female-specific risk factors

Several sex-specific biological differences and risk factors contribute to CAD in women. Women have smaller epicardial arteries than men, even after accounting for body size and left ventricular mass [10]. They also have higher coronary blood flow, which may cause lower endothelial shear stress and could partially explain lower CAD rates in women when stratified for age [11]. A factor theorized as to why CAD rates in women substantially increase postmenopausal is decreased endogenous estrogen supply. Estrogen downregulates the renin-angiotensin-aldosterone system (RAAS) as well as fibrinolytic pathways, which play a pivotal role in the development of CAD and hypertension; however, in postmenopausal women, this protective role is diminished. This sharp decline in estrogen is also associated with an increased risk of obesity and dyslipidemia [12].

In 2018, a Presidential Advisory from the American Heart Association (AHA) and the American College of Obstetricians and Gynecologists emphasized that adverse pregnancy outcomes (APOs), including gestational DM, preeclampsia, and preterm delivery, are often overlooked as CAD risk factors in traditional CAD risk assessment tools such as the Framingham Risk Score [13]. Moreover, the impact of shared risk factors such as hypertension and DM is not adequately adjusted for in women. Recent studies, however, have highlighted the importance of APOs as risk factors. In 2024, Venkatesh et al. found that APOs were associated with an elevated 30-year atherosclerotic cardiovascular disease risk [14]. Similarly, a 2023 national cohort study by Crump et al. involving over two million women found that multiple APOs are independently associated with increased ischemic heart disease incidence, even up to 46 years postpartum [15]. These findings underscore the importance of integrating obstetric history in both risk assessment and prevention of CAD (Tab. 1).

**Table 1:** Recent Studies Identifying the Increased Risk of Coronary Heart Disease due to Adverse Pregnancy Outcomes

Study	Study Population	Summary of Outcomes
Venkatesh et al., [14]	4,273 women between the ages of 15–44 with known APOs	APOs were associated with elevated 30-year ASCVD risk measured at 2–7 years after delivery. The magnitude of risk was higher with a greater number of APOs experienced, and with gestational DM
Crump et al. [15]	A national cohort study of 2,195,266 women with first singleton delivery, excluding women with previous ischemic heart disease and missing antenatal information	All five APOs studied were independently associated with increased incidence of ischemic heart disease

*APO* Adverse Pregnancy Outcomes; *DM* Diabetes Mellitus; *ASCVD* Atherosclerotic Cardiovascular Disease; *CAD* Coronary Artery Disease

**Fig. 2:**
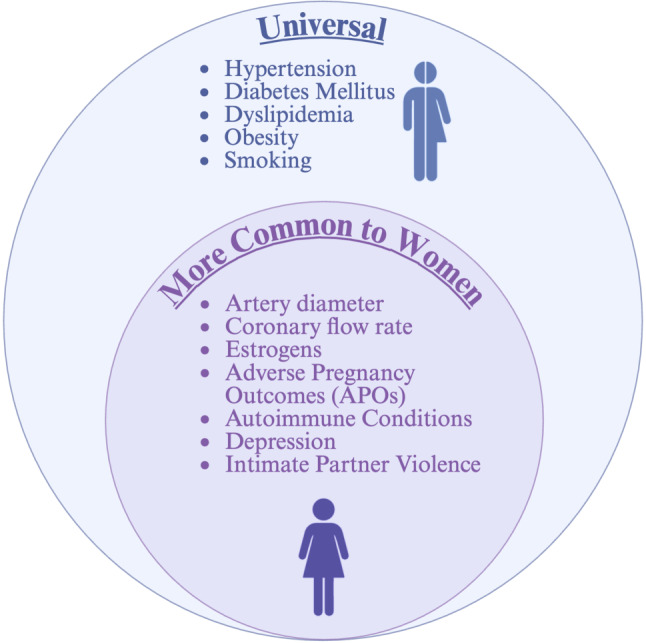
Risk Factors for Coronary Artery Disease That are Shared and More Common to Women

While not sex-specific, autoimmune conditions are substantially more prevalent in women than men [16]. Chronic inflammatory states increase endothelial dysfunction, and chronic steroid treatment increases the risk of hyperglycemia and dyslipidemia, which also contribute to CAD development [17]. Other risk factors disproportionately impacting women are psychosocial. Depression is more common in women and is a recognized risk factor for incident MI and cardiac death [18]. Similarly, intimate partner violence impacts 27% of women at some point in their lives globally, and the chronic stress it induces, even years after the abuse stops, is associated with increased rates of dyslipidemia, obesity, smoking, and cardiovascular events [19]. Psychological distress has been shown to impact the hypothalamic-pituitary-adrenal axis and autonomic nervous system. This can cause cardiac autonomic dysregulation, trigger coronary vasoconstriction, and increase oxygen demand, which can precipitate angina symptoms. It also contributes to medication non-adherence and unhealthy lifestyle choices [20]. It is now abundantly evident that efforts to recognize and address mental distress in women are necessary to reduce the overall burden of CAD (Fig. 2).

## Clinical presentation and management

The European Society of Cardiology recommends that, unless a noncardiac cause is evident, all patients presenting with chest pain should undergo initial evaluation with an electrocardiogram and cardiac biomarkers [21]. However, in a pivotal systematic review and meta-analysis of over one million patients by van Oosterhout et al. comparing symptom presentation in acute coronary syndrome (ACS) between genders, women have lower odds of ‘typical symptoms’ like chest pain and diaphoresis, and higher odds of presenting with ‘atypical symptoms’ like nausea or vomiting, dyspnea, syncope, fatigue, dizziness, jaw pain, neck pain, and interscapular pain (Tab. 2; [22]). Similarly, Araujo et al. reported that women presenting with ACS often report higher pain intensity, referred pain, and other symptoms rather than pain compared to men [23]. These findings highlight the diverse manifestations of ACS in women, and clinicians should move beyond the traditional perception of the condition.

**Table 2:** Results of Meta-analysis of the Adjusted Odds Ratio and 95% Confidence Interval of Certain Acute. Coronary Syndrome Symptoms Experienced by Women Relative to Men as Reported by van Oosterhout et al. [22]

Symptoms in Acute Coronary Syndrome	Adjusted OR of symptoms experienced by women relative to men [Corresponding 95 CI]	Heterogeneity % (I2)
Chest pain	0.67 (0.62–0.73)	72%
Diaphoresis	0.75 (0.72–0.78)	28%
Shortness of breath	1.22 (1.01–1.46)	0%
Nausea or vomiting	1.63 (1.21–2.19)	0%
Fatigue	1.34 (0.94–1.90)	0%
Interscapular man	1.89 (1.27–2.82)	0%
Dizziness or Lightheadedness	1.41 (0.96–2.07)	0%
Neck pain	1.71 (1.00–2.93)	0%
Palpitations	1.91 (0.91–4.00)	0%
Jaw pain	1.67 (1.01–2.78)	0%

*OR* Odds Ratio; *CI* Confidence Interval

Recent studies have highlighted that mainstream CAD risk assessment tools often underestimate the risk in female patients with chest pain, which can result in delays in seeking care and initiating appropriate diagnostic workup [13, 24]. Emergency department data have shown that female patients with chest pain are more likely to be categorized as having a low pretest probability of ACS, making them less likely to undergo coronary angiography and contributing to a prolonged “door-to-balloon time” [25, 26]. One strategy to improve ACS detection in women is to use gender-specific cardiac biomarker thresholds. Elevated high-sensitivity cardiac troponin levels are associated with a greater risk of developing cardiovascular disease and major cardiovascular events in women compared to men [27, 28]. Compared to contemporary assays, a high-sensitivity cardiac troponin assay with a sex-specific diagnostic threshold has been shown to increase the diagnosis of MI in women from 11 to 22%, versus from 19 to 21% in men [29].

While guidelines recommend similar noninvasive testing strategies for men and women with stable CAD, some modalities offer greater utility for conditions disproportionately affecting women. Cardiac magnetic resonance with quantitative perfusion techniques and positron emission tomography with myocardial perfusion imaging are highly accurate for diagnosing coronary microvascular dysfunction (CMD), which will be discussed further below [30]. Similarly, coronary computed tomography angiography (CCTA) is effective in detecting non-obstructive CAD and can provide valuable information in risk stratification [30].

### Medication prescription and adherence disparities

Optimal medical therapy for CAD management is well established, yet its implementation for women remains inadequate. Analysis of national registry data in the United States found that women were less likely to receive statin therapy or receive it at its guideline-recommended intensity after an MI [31]. Nonacceptance of statin therapy and less than optimal LDL-cholesterol control are also more common in women, especially in those whose secondary language was English [32]. Another study similarly reported that women are less frequently prescribed and adherent to antiplatelet medications [33]. Likewise, in a systematic review and meta-analysis involving more than two million patients, Zhao et al., found that women at high risk for or diagnosed with CAD were prescribed ACE inhibitors and beta-blockers less often than men [34]. These findings call for the need for increased access to these treatment options and enhanced counseling of female patients. However, it must be noted that some data suggest that these disparities may be overstated and partially due to female patients presenting at an older age. An age-stratified analysis after percutaneous coronary intervention for patients presenting with STEMI revealed no statistically significant differences between males and females in clinical outcomes at 1‑month and 1‑year follow-up [40]. Another study that suggested female patients in China presenting with non-STEMI were less likely to receive aspirin or invasive management was also corrected with age-stratification [32]. Thus, age appears to be a confounding factor in assessing gender-based disparities in CAD, highlighting the need for age-stratified analysis in future research.

## Non-obstructive CAD and spontaneous coronary artery dissection

Although distinct, non-obstructive CAD and spontaneous coronary artery dissection (SCAD) are key considerations in women presenting with chest pain. Non-obstructive CAD includes pathologies in which cardiac catheterization reveals no significant coronary obstruction, including angina with no obstructive coronary artery disease (ANOCA), ischemia with no obstructive coronary artery disease (INOCA), and myocardial infarction with non-obstructive coronary arteries (MINOCA) [35]. While ANOCA presents with chest pain, it does not have objective evidence of ischemia. The underlying mechanisms of these conditions include CMD, coronary artery vasospasm, oxidative stress, hypercoagulable states, and nonobstructive atherosclerosis [35, 36]. CMD often causes anginal symptoms at rest, further complicating the diagnosis of chest pain. The predominance of women with these conditions and the reliance on “stenosis-centric” diagnostic strategies like CCTA contribute to underdiagnosis and undertreatment of heart disease in women, particularly those with non-obstructive CAD. A diagnostic approach that incorporates functional tests, which assess factors beyond epicardial disease, can support the identification and management of these conditions.

Management of non-obstructive CAD involves a multifaceted approach [36]. Lifestyle modifications include diet, weight loss, exercise, stress management, and smoking cessation. Risk factor management includes controlling hypertension, dyslipidemia, and DM. Pharmacologic therapy centers around an anti-anginal strategy consisting of beta blockers, calcium channel blockers, and the anti-anginal nicorandil. In addition, treatment with statins can improve endothelial function, while ACE inhibitors may improve microvascular function by preventing vascular remodeling [36]. Long-acting nitrates, often effective for anginal chest pain in obstructive CAD, are less effective in INOCA, particularly due to CMD. This is thought to be due to its effect primarily on epicardial coronary arteries and due to potentially worsening endothelial dysfunction through oxidative stress. Emerging evidence also highlights the role of the autonomic nervous system and visceral pain processing in INOCA. One pilot study found that 62% of women with INOCA displayed “central sensitization”, indicating a heightened pain response mediated by the central nervous system [37]. This suggests that, beyond impaired myocardial blood flow, the perception of angina in INOCA may be influenced by abnormal autonomic regulation and neural processing of pain signals. Further research may provide invaluable insights into heart disease presentations across diverse populations.

**Fig. 3:**
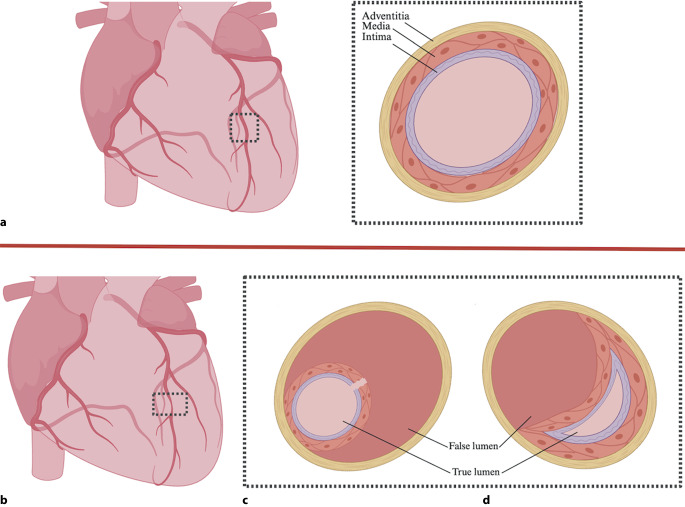
Spontaneous Coronary Artery Dissection (SCAD). **a** Heart With Normal Coronary Arteries and Cross-section of Normal Coronary Artery. **b** Coronary Artery Affected by SCAD. **c** Diagram Depicting Intraluminal Hemorrhage, a Proposed Mechanisms for SCAD. **d** Diagram Depicting Intimal Hemorrhage, a Mechanisms for SCAD

SCAD is caused by a non-traumatic, non-atherosclerotic separation of coronary artery layers leading to myocardial ischemia (Fig. 3). Strikingly, it accounts for 1–4% of all ACS; 90% of cases occur in women, and it is responsible for up to 35% of MI in women under 50 [38]. It is also the most common cause of pregnancy-associated MI [39]. SCAD patients lack traditional CAD risk factors and are frequently associated with emotional stressors, hormonal fluctuations, and connective tissue disorders such as fibromuscular dysplasia. Compared to classic CAD, conservative management with observation is generally advised for clinically stable patients, as most dissections resolve on their own, and percutaneous coronary intervention can present technical difficulties and less favorable procedural results. Conservative management is also recommended for pregnancy-related SCAD. These patients should also be counseled on avoiding strenuous exercise, managing comorbidities, and the impact of hormonal fluctuations such as pregnancy, on precipitating disease [38, 39].

## Future directions

### Increasing awareness in patients and addressing under-representation

Enhancing CAD diagnosis and treatment in women will remain limited until female patients are more aware of their own risk. AHA survey data analysis from 2009 to 2019 revealed decreased awareness amongst women regarding cardiovascular disease as their leading cause of death, especially in Hispanic, non-Hispanic, Black, and young women, the latter being a group in which primordial/primary prevention may be most effective [40]. Further initiatives in female patient education regarding the risk of CAD to enhance health literacy regarding cardiovascular screening studies for CAD, CAD treatment options, CAD treatment adherence, and overall lifestyle modifications will be critical. Similarly, there has been a historic under-representation of female participants in CAD research. A review of clinical trials involving non-STEMI patients between 1994 to 2010 revealed that less than 33% of trial participants were women [41]. Similarly, Jin et al. analyzed 740 cardiovascular trials completed between 2010–2017 and assessed that women were underrepresented relative to disease prevalence in both coronary heart disease and ACS trials [42]. Various socioeconomic, racial, and cultural factors may prohibit female participation and affect their adherence to medical therapies [43]. Increasing female involvement in clinical trials and powering them for sex-specific analyses will be imperative to discover biological variance in pathophysiology and to ensure appropriate management strategies for females. Policy-level interventions could include mandating sex-specific enrollment in future studies with sufficient statistical power, as well as providing logistical support—such as transportation, childcare, and flexible scheduling—to facilitate participation. Future directions should also include greater involvement of multidisciplinary women’s health programs to assist in increasing accessibility for women to specialized care, accurately recognizing signs and symptoms of CAD in women, offering specialized testing, and providing women support in various avenues, including psychological, emotional, and social health [44].

### Revisiting hormonal replacement therapy

In the early 2000s, the publication of the Women’s Health Initiative (WHI) study reported prohibitive cardiovascular risks associated with hormone replacement therapy (HRT), leading to a significant decline in its use [45]. However, recent studies have reevaluated its potential, resulting in a more nuanced understanding of its applicability, particularly for cardiovascular diseases. Age-stratified analysis of the WHI study revealed that the absolute risks of adverse events with HRT were significantly lower in women aged 50–59 and those who initiated HRT within ten years of menopause, giving credence to the “timing hypothesis”, which suggests that the cardiovascular disease risk of HRT is contingent on the timing of its initiation relative to menopause [46]. The “timing hypothesis” has been concordant with other publications, particularly a large meta-analysis of 19 randomized controlled trials with over 40,000 postmenopausal women that found those who initiated HRT within 10 years of menopause had a significantly reduced relative risk of 0.52 of coronary heart disease compared to placebo or no treatment [47].

Presently, HRT is recommended for those with unmanageable postmenopausal symptoms as well as for the prevention of bone loss/fractures [48]. However, it is contraindicated in those with a history of breast cancer, coronary heart disease, previous venous thromboembolic event or stroke, active liver disease, undiagnosed abnormal genital bleeding, and in those with known or suspected estrogen-dependent neoplasia. Adverse effects include increased risk of venous thromboembolism, breast cancer, endometrial cancer when estrogen is used unopposed in women with a uterus, and dementia (Tab. 3). Currently, HRT is not recommended by professional cardiology societies, for primary or secondary prevention of cardiovascular disease. However, with growing evidence suggesting the potential of early HRT initiation in the primary prevention of CAD, newer guidelines may evolve.

**Table 3:** Current Food Drug Administration Approved Indications, Contraindications, and Adverse Effects of Oral or Transdermal Hormone Replacement Therapy per the 2022 Hormone Therapy Position Statement of the North American Menopause Society [48]

FDA Approved Indications	Contraindication	Adverse Effects
Moderate to severe VMS	Unexplained vaginal bleeding	Increased risk for VTE
Prevention of bone loss	Liver disease	Increased risk for gallbladder disease
Premature hypoestrogenism	Prior Estrogen-sensitive cancer	Increased risk for stroke
Genitourinary syndrome	Prior coronary heart risease, stroke, MI, or VTE	Increased risk for breast cancer
	Personal history or inherited high risk of thromboembolic disease	If Estrogen is enadequately opposed, increased risk of endometrial hyperplasia and endometrial cancer

*FDA* Food Drug Administration; *VMS* vasomotor symptoms; *MI* myocardial infarction; *VTE* venous thromboembolism

## Implementing artificial intelligence in assessment and the potential of epigenetic modifications

The use of artificial intelligence (AI) to facilitate a comprehensive cardiovascular assessment (including CAD) in female patients will become a growing practice to analyze large volumes of data on primordial, primary, and secondary prevention of heart disease in women. For primordial prevention, AI may analyze data from various applications, including activity and diet trackers, fitness applications, and digital scales, to study the potential development of CAD risk factors, including hypertension, dyslipidemia, and obesity. Similarly, AI can be used in primary prevention through screening women who are at risk for but not diagnosed with the conditions already mentioned. Lastly, secondary prevention could be achieved via AI algorithms to identify women with CAD who would still benefit from further intensive cardiac therapies, including but not limited to aggressive lifestyle modifications, medication eligibility, and monitoring of disease status [49].

While further research is needed on this topic, sex differences in DNA methylation patterns have been observed in genes and pathways such as PLA2G7 and KDMA6 relevant to cardiometabolic health [50]. These differences may contribute to sex-specific variations in CAD, including risk, presentation, and outcomes. However, the AHA has noted that findings of sex-specific methylation in these genes, while intriguing, require validation in larger, adequately powered studies before robust conclusions can be drawn. Additional research in this area will be vital to determine their true significance.

## Conclusion

Despite efforts by organizations like The Lancet Commission, AHA, and the European Society of Cardiology, gender disparities in CAD remain, requiring ongoing action to close the gap. Clinical studies evaluating if stricter goals are warranted for women with modifiable risk factors, further analysis of the role of estrogen in atherosclerotic events and in primary prevention, and continued education on symptomatology are necessary both for patients and healthcare providers. Risk stratification of patients, including gender-specific attributes, is also needed, and the potential of AI in identifying those at risk should be embraced globally. Clinical trials must be more inclusive for women moving forward to identify variation in disease pathophysiology and potential management strategies. At the societal level, it is important to identify, address, and manage psychosocial factors that may contribute to CAD in women. Only through a concerted, multidimensional approach can we hope to achieve true equity in the prevention, diagnosis, and management of CAD for women worldwide.
